# Joint and interactive associations of nutritional risk and pulmonary infection with tracheostomy decannulation in neurorehabilitation inpatients: a retrospective cohort study

**DOI:** 10.3389/fnut.2026.1944451

**Published:** 2026-10-07

**Authors:** Jiang Wang, Shuguang Liang, Huizhen Zhang, Xin'ao Wang, Shuya Yuan, Liugen Wang, Jing Zeng, Shu Yan, Ju Huang

**Affiliations:** 1 Rehabilitation Department, Jintan District Hospital of Traditional Chinese Medicine, Changzhou, Jiangsu, China; 2 Department of Rehabilitation Medicine, First Affiliated Hospital of Zhengzhou University, Zhengzhou, China; 3 School of Public Health, Zhengzhou University, Zhengzhou, China

**Keywords:** cox regression, decannulation, interaction, neurorehabilitation, nutritional risk, pulmonary infection, tracheostomy

## Abstract

**Background:**

Nutritional risk and pulmonary infection are common in tracheostomized patients entering neurorehabilitation. Their coexistence may combine limited physiological reserve with respiratory instability, but evidence on their joint association and interaction with decannulation is limited.

**Methods:**

This retrospective cohort included 884 tracheostomized adults with neurological disorders admitted to a tertiary rehabilitation department between January 2020 and May 2025. Nutritional risk was defined as a Nutritional Risk Screening 2002 score ≥3, and active pulmonary infection was assessed at rehabilitation admission. Successful decannulation within 30 days was the event. Cox models were used to explore individual and joint associations and multiplicative interaction. The primary adjusted model was adjusted for demographic characteristics, comorbidity, neurological severity, and acute-care trajectory. An extended model additionally included concurrent respiratory-support, airway-protection, secretion-management, and care-related characteristics. Fifteen-day, pre-rehabilitation timing, and censoring sensitivity analyses were performed. Adjusted 15-and 30-day decannulation probabilities and 30-day restricted mean time remaining tracheostomized were also estimated.

**Results:**

Overall, 574 patients (64.9%) were successfully decannulated within 30 days. Kaplan–Meier probabilities of decannulation were 95.4% with neither condition, 82.2% with pulmonary infection alone, 85.6% with nutritional risk alone, and 49.5% with both conditions. In the primary adjusted model, the hazard ratios (HRs) were 0.619 [95% confidence interval (CI), 0.475–0.806] for pulmonary infection alone, 0.642 (95% CI, 0.483–0.853) for nutritional risk alone, and 0.203 (95% CI, 0.157–0.262) for both conditions. The multiplicative interaction was significant (interaction HR, 0.510; 95% CI, 0.356–0.731; *P* < 0.001). Adjusted 30-day decannulation probabilities were 94.9%, 85.0%, 86.0%, and 47.6%, respectively. The dual-positive phenotype had a 30-day restricted mean time remaining tracheostomized of 22.8 days versus 10.5 days with neither condition, and the adjusted 30-day additive interaction contrast was −28.5 percentage points (95% CI, −35.2 to −15.8). Findings were similar in the extended, 15-day, timing-adjusted, and censoring sensitivity analyses.

**Conclusions:**

Nutritional risk and active pulmonary infection at rehabilitation admission were each associated with a lower rate of successful decannulation. Their coexistence showed a stronger joint association on the multiplicative hazard scale and an adverse departure from additivity on the 30-day absolute probability scale. These findings support combined nutritional and respiratory assessment.

## 1 Introduction

Many patients with severe neurological disorders remain tracheostomized when they enter rehabilitation (1). Successful decannulation is an important step in neurorehabilitation (1, 2). It may reduce the burden of airway management and support swallowing, communication, and functional recovery (3). In clinical practice, safe removal of the tracheostomy tube typically requires stable spontaneous breathing, upper-airway patency, effective cough, and adequate secretion control (1, 3–5). Since these functions may recover at different rates, decannulation readiness should be assessed across multiple domains (1). Premature removal may be unsafe, whereas unnecessary delay prolongs dependence on an artificial airway (1–3). Therefore, it is important to identify the appropriate time for decannulation.

Patients entering neurological rehabilitation are also vulnerable to nutritional risk. Dysphagia, impaired consciousness, feeding dependence, and limited food intake may coexist with inflammation and increased metabolic demand (6, 7). In this context, nutritional risk may reflect limited physiological reserve rather than only low body weight (8). Reduced muscle mass and strength may affect expiratory muscle performance and cough effectiveness, both of which are relevant to airway clearance and decannulation readiness (9, 10). Inadequate nutritional intake may also reduce the patient's capacity to participate actively in rehabilitation (11). Although previous decannulation studies have considered nutritional indicators such as body mass index and serum albumin, standardized nutritional screening tools have been less frequently examined (10, 12). More importantly, nutritional risk may not act alone, and its association with decannulation may become more pronounced when other adverse clinical conditions coexist.

Pulmonary infection is another important concern. Active infection may increase secretion burden, compromise oxygenation, and reduce respiratory stability. These effects can delay progression through a multidomain decannulation-readiness process that also considers airway patency, cough effectiveness, secretion management, and tolerance of tube occlusion or capping (1, 3, 13). In a cohort of 324 patients with moderate-to-severe traumatic brain injury, pneumonia was associated with an estimated 24.4-day increase in time to decannulation (14). Consistently, a recent systematic review identified the absence of pulmonary infection as a predictor of successful decannulation after acquired brain injury (10). However, infection is also related to illness severity, airway protection, and functional status. Therefore, its association with decannulation should be interpreted within the broader clinical recovery process and assessed after multivariable adjustment.

Nutritional risk and pulmonary infection may be mutually affected and exacerbate adverse clinical conditions surrounding decannulation (15, 16). Infection-related inflammation can suppress appetite and increase catabolic demand, thereby worsening nutritional status (15). In turn, limited nutritional and muscle reserve may impair respiratory muscle function and cough effectiveness, reducing airway clearance and slowing recovery from infection (16). Therefore, their coexistence may represent a particularly vulnerable clinical phenotype characterized by both respiratory instability and reduced capacity for secretion clearance and rehabilitation (1, 11, 16). Previous decannulation studies have generally evaluated nutritional indicators and pulmonary infection separately or treated them as independent variables in multivariable models (10, 17). It remains insufficiently understood whether their coexistence is associated with a further reduction in decannulation outcomes beyond their separate effects.

Based on this, the current study retrospectively analyzed routinely collected clinical data from tracheostomized patients entering neurorehabilitation. We explored the individual and joint associations of nutritional risk and active pulmonary infection at rehabilitation admission with time to successful decannulation, tested their interaction on the multiplicative hazard scale, and additionally quantified absolute 15- and 30-day decannulation outcomes.

## 2 Methods

### 2.1 Procedures and participants

This retrospective cohort study included tracheostomized neurological rehabilitation inpatients at the First Affiliated Hospital of Zhengzhou University between January 2020 and May 2025. The study-specific protocol for retrospective secondary analysis of these records was approved by the Ethics Committee of the First Affiliated Hospital of Zhengzhou University in 2025 (approval No. 2025-KY-1228-002). Since 2020, as part of routine hospitalization procedures, patients with decision-making capacity, or their legally authorized representatives when appropriate, had provided written authorization permitting the de-identified use of their clinical information for research purposes. This general authorization preceded and was not specific to the present retrospective analysis. No additional study-specific written informed consent was required for the present retrospective secondary analysis.

All consecutive eligible patients during the study period were included, without sampling or selective case entry. The inclusion criteria were as follows: (1) age ≥18 years; (2) diagnosis of stroke, traumatic brain injury, or another neurological disease or injury; (3) tracheostomy for the current neurological condition or related acute complications, with the tube still in place at rehabilitation admission; (4) stable vital signs at rehabilitation admission; and (5) availability of sufficient information to ascertain the key exposures, outcome, and follow-up time. The methods used to address missing data are described in the Statistical Analysis section. The exclusion criteria were as follows: (1) decannulation before completion of nutritional and infection assessments at admission; (2) long-term dependence on a tracheostomy tube before the onset of the current neurological condition; and (3) obvious logical inconsistencies in the medical records that could not be resolved after review of the original records.

Data were extracted from the electronic medical record system. For cases in which the information could not be directly retrieved, two researchers independently reviewed the medical records and manually extracted the relevant details. Any discrepancies were resolved through verification of the original records or adjudication by a third researcher. For patients with multiple hospital admissions, only the first eligible admission was included to ensure that each patient contributed only one observation period. The sample size was estimated based on previous literature, with further details provided in Supplementary material S1 (6, 18–21). The calculation indicated that at least 802 evaluable patients were required. After allowing for approximately 5% of screened records with unavailable or irreconcilable key information, the target number of screened records was 845. Based on the available departmental caseload, extending the study window back to January 2020 was expected to yield the required number of records.

After admission to the department of rehabilitation, all patients received individualized multidisciplinary rehabilitation according to their consciousness level, respiratory status, and functional capacity. The rehabilitation program generally included tracheostomy and respiratory management, nutritional and feeding management, swallowing-related care, motor rehabilitation, and medical management of pulmonary infection and other comorbidities. Airway management included humidification, positioning, suctioning, cuff management, and assisted secretion clearance. Patients who were sufficiently alert to follow commands additionally received voluntary cough and airway-clearance training. Motor rehabilitation consisted of passive or active exercises and activities of daily living training, as tolerated. No study-specific rehabilitation intervention was conducted.

### 2.2 Assessments

#### 2.2.1 Nutritional risk and pulmonary infection

Nutritional risk and pulmonary infection were the key exposures in this study, and both were assessed within 12 h of rehabilitation admission. Nutritional Risk Screening 2002 (NRS-2002) was part of routine nutritional screening at rehabilitation admission and was completed by trained clinical staff. Throughout the study period, the same admission NRS-2002 procedure was used. The disease-severity component was scored according to the standard NRS-2002 rubric for current illness severity and associated metabolic stress. Impaired nutritional status and disease severity were each scored from 0 to 3 points, with 1 additional point for patients aged ≥70 years. The total score could range from 0 to 7 (8). A score ≥3 defined nutritional risk, consistent with the intended screening purpose of NRS-2002, and was not interpreted as a diagnosis of malnutrition. Screening entries were obtained from the standardized admission assessment and were cross-checked against source medical records when incomplete or internally inconsistent.

The disease-severity component represented the highest applicable NRS-2002 disease-severity category among concurrent clinical conditions instead of the sum of scores for individual diagnoses. The admission screening record retained the component score but did not separately identify which diagnosis determined that category. Therefore, we therefore performed a review to determine whether active pulmonary infection resulted in a higher disease-severity category than would have been assigned from the underlying neurological condition alone.

Pulmonary infection status was recorded at rehabilitation admission, defined as an active lower respiratory tract infection documented by the treating physician and supported by compatible chest imaging together with contemporaneous clinical or laboratory evidence, following the principles of the Chinese guideline for hospital-acquired and ventilator-associated pneumonia (22). In routine practice, suspicion of infection was prompted by respiratory or systemic features such as fever, increased or purulent airway secretions, abnormal pulmonary auscultation, or worsening oxygenation, followed by chest radiography or computed tomography and laboratory assessment. Compatible imaging findings were required for classification and included new, progressive, or unresolved infiltrative, consolidative, or ground-glass abnormalities considered clinically consistent with active pulmonary infection. Leukocyte count and inflammatory markers were used as supportive evidence, and microbiological findings were considered supportive when available. A positive airway culture, isolated atelectasis, or a residual radiographic abnormality was not considered sufficient on its own to classify a patient as having active pulmonary infection. Patients already receiving treatment for an infection diagnosed during acute care were classified as infected only when the infection remained clinically active at rehabilitation admission.

#### 2.2.2 Decannulation of the tracheostomy tube

Decannulation was considered after the primary neurological condition and vital signs had stabilized and invasive ventilatory support was no longer required. Throughout the study period, readiness was assessed using the same core clinical domains, including respiratory stability, airway patency, cough effectiveness, secretion burden and suctioning requirements, oxygenation, and tolerance of tracheostomy-tube capping when applicable (1, 23). Active pulmonary infection did not constitute an automatic permanent contraindication to decannulation. Instead, adequate control of infection and respiratory stability were components of the overall readiness assessment. Control of pulmonary infection was judged clinically from improvement or resolution of acute infectious features, stable oxygenation without increasing oxygen requirements, stable or improving airway secretions, and no evidence of progressive infection requiring escalation of antimicrobial or respiratory support. The treating attending physician could proceed with decannulation after review and approval by a senior physician at the rank of chief physician. Decannulation proceeded if the patient remained clinically stable during the capping trial without respiratory distress, evidence of upper-airway obstruction, or deterioration in oxygenation. Airway endoscopy or imaging was performed when clinically indicated. Oral and tracheal secretions were suctioned before tube removal under pulse oximetry and cardiac monitoring. Successful decannulation was defined as removal of the tracheostomy tube without the need for re-cannulation within 72 h. Patients decannulated within the 30-day window were observed for at least 72 h after tube removal, and event time was recorded as the date of tube removal.

The core rehabilitation assessment and decannulation-readiness framework remained consistent throughout the 2020–2025 study period, although individual treatment decisions were tailored to each patient's clinical condition.

#### 2.2.3 Covariates

Covariates were extracted from rehabilitation-admission assessments and the preceding acute-care records. Operational definitions and coding rules for disease category, neurological severity, acute-care exposure, airway-related characteristics, and caregiver status are provided in Supplementary material S2.

### 2.3 Statistical analysis

Continuous variables were presented as means and standard deviations or medians and interquartile ranges (IQRs), as appropriate. Categorical variables were presented as frequencies and percentages. Time zero was rehabilitation admission, and successful tracheostomy decannulation was the event. Follow-up continued until successful decannulation or the earliest of discharge, transfer, death, and day 30. Patients without decannulation were right-censored at the corresponding date. The cumulative probability of decannulation was estimated using the Kaplan–Meier method. Median time to decannulation was reported when estimable, and numbers at risk were tabulated at prespecified time points.

Cox proportional-hazards models were used to estimate the individual and joint associations of nutritional risk and pulmonary infection with decannulation. The group with neither condition was the reference. Model 1 included nutritional risk, pulmonary infection, and their product term. Model 2, the primary adjusted model, additionally included age, sex, smoking history, Charlson Comorbidity Index (24). Glasgow Coma Scale (GCS) (25). Barthel Index (26). brainstem or posterior fossa involvement, pre-tracheostomy intubation duration, ICU length of stay, and early tracheostomy. Model 3 was an extended clinical model that further included mechanical ventilation at rehabilitation admission, swallowing reflex, gag reflex, cough ability, suctioning frequency, and use of a privately hired caregiver. Disease category was used as a stratification factor in adjusted models, and tied events were handled using the Efron method (27). Hazard ratios (HRs) and 95% confidence intervals (CIs) were reported; HR < 1 indicated a lower rate of successful decannulation. Multiplicative interaction was assessed from the nutritional-risk-by-pulmonary-infection product term and by likelihood-ratio testing of models with and without the interaction.

Several sensitivity and supplementary analyses were performed. First, the 30-day primary analysis was repeated with follow-up administratively truncated at 15 days. Second, to account for stage of recovery before rehabilitation admission, a timing-adjusted sensitivity model added the neurological-onset-to-rehabilitation and tracheostomy-to-rehabilitation intervals to Model 2. Third, because censoring caused by discharge or transfer could be informative, a sensitivity analysis assumed that every patient censored before day 30 remained non-decannulated through day 30. Fourth, standardized adjusted probabilities of successful decannulation at 15 and 30 days were obtained from Model 2 by setting each participant to each of the four exposure phenotypes and averaging predicted probabilities over the observed covariate distribution. The corresponding 30-day restricted mean time remaining tracheostomized was calculated as the area under the standardized survival curve through day 30 (28). Departure from additivity on the absolute probability scale was summarized as P11–P10–P01+P00, where P11 denotes the standardized decannulation probability with both exposures, P10 nutritional risk only, P01 pulmonary infection only, and P00 neither condition. The 95% CIs were obtained from parametric simulation based on the fitted-model coefficient covariance matrix (29). Finally, Model 2 was repeated after excluding the 19 patients without a preceding endotracheal-intubation episode. Additionally, as pulmonary infection contributed to the NRS-2002 disease-severity component in a small subset of patients, an sensitivity analysis replaced NRS-2002-defined nutritional risk with the impaired-nutritional-status component alone. Nutritional-status impairment was defined as a component score ≥1 and did not incorporate either the disease-severity component or the age point. The same Model 2 adjustment set was used.

Exploratory subgroup analyses compared the dual-positive phenotype with all other phenotypes within disease categories and admission Glasgow Coma Scale strata (3–8, 9–12, and 13–15); these contrasts were not interpreted as subgroup-specific replications of the formal interaction. Heterogeneity was assessed using interaction terms and likelihood-ratio tests. The proportional-hazards assumption was assessed using scaled Schoenfeld residuals (30). When time dependence was detected for adjustment covariates, a time-split sensitivity analysis allowing their coefficients to differ after day 15 was used to assess whether the primary exposure estimates materially changed.

Patients with missing decannulation outcome, nutritional-risk status, or pulmonary-infection status were excluded because these were key variables. Before imputation, missingness for individual covariates did not exceed 7% overall and was summarized by exposure phenotype in Supplementary material S3. Remaining covariate missingness was handled using multiple imputation by chained equations with 30 imputed datasets (31, 32). The imputation model included the exposures, adjustment variables, event indicator, and survival-time information, and estimates were combined using Rubin's rules. All tests were two-sided, with *P* < 0.05 considered statistically significant. Analyses were performed using Python 3.12.13. Reporting followed the Strengthening the Reporting of Observational Studies in Epidemiology (STROBE) checklist (33).

## 3 Results

### 3.1 Participant information

A total of 917 records were screened. Thirty-three were excluded: 10 because decannulation occurred before completion of the admission nutritional and infection assessments, 7 because tracheostomy dependence predated the current neurological condition, and 16 because key exposure, outcome, follow-up, or internally inconsistent information could not be resolved after record review. The final cohort included 884 patients, exceeding the preset sample-size requirement. The mean age was 55.9 ± 13.7 years, and 641 patients (72.5%) were men. Hemorrhagic stroke was the most common primary diagnosis (469, 53.1%), followed by ischemic stroke (126, 14.3%), traumatic brain injury (115, 13.0%), and other neurological conditions (174, 19.7%). Nutritional risk was present in 574 patients (64.9%), and pulmonary infection was present in 624 (70.6%). The four mutually exclusive phenotypes comprised 150 patients (17.0%) with neither condition, 160 (18.1%) with pulmonary infection alone, 110 (12.4%) with nutritional risk alone, and 464 (52.5%) with both conditions. Baseline characteristics are shown in Table 1.

**Table 1:** Baseline characteristics according to nutritional risk and pulmonary infection phenotype.

Characteristic	Overall (*n* = 884)	Neither (*n* = 150)	Infection only (*n* = 160)	Nutritional risk only (*n* = 110)	Both (*n* = 464)
Demographic and nutritional characteristics					
Age, years	55.9 ± 13.7, *n* = 884	50.6 ± 14.3, *n* = 150	54.7 ± 15.1, *n* = 160	56.4 ± 10.9, *n* = 110	57.9 ± 13.2, *n* = 464
Male sex, *n* (%)	641 (72.5)	117 (78.0)	114 (71.2)	92 (83.6)	318 (68.5)
Smoking history, *n*/*N* (%)	267/873 (30.6)	31/147 (21.1)	39/159 (24.5)	25/108 (23.1)	172/459 (37.5)
Missing, *n* (%)	11 (1.2)	3 (2.0)	1 (0.6)	2 (1.8)	5 (1.1)
Disease category					
Hemorrhagic stroke, *n* (%)	469 (53.1)	91 (60.7)	89 (55.6)	78 (70.9)	211 (45.5)
Ischemic stroke, *n* (%)	126 (14.3)	20 (13.3)	21 (13.1)	13 (11.8)	72 (15.5)
Traumatic brain injury, *n* (%)	115 (13.0)	15 (10.0)	29 (18.1)	10 (9.1)	61 (13.1)
Other neurological conditions, *n* (%)	174 (19.7)	24 (16.0)	21 (13.1)	9 (8.2)	120 (25.9)
Clinical severity and acute-care exposure					
Charlson Comorbidity Index, points	2.0 [2.0, 3.0], *n* = 871	2.0 [1.0, 3.0], *n* = 147	2.0 [1.0, 3.0], *n* = 159	2.0 [2.0, 2.0], *n* = 106	2.0 [2.0, 3.0], *n* = 459
Glasgow Coma Scale, points	8.0 [6.0, 10.0], *n* = 876	8.0 [8.0, 13.0], *n* = 150	8.0 [6.0, 10.0], *n* = 158	8.0 [8.0, 9.0], *n* = 108	7.0 [5.0, 9.0], *n* = 460
Barthel Index, points	0.0 [0.0, 15.0], *n* = 864	5.0 [0.0, 40.0], *n* = 146	5.0 [0.0, 15.0], *n* = 158	5.0 [0.0, 5.0], *n* = 108	0.0 [0.0, 10.0], *n* = 452
ICU length of stay, days	12.0 [6.0, 19.0], *n* = 826	10.0 [5.0, 15.0], *n* = 136	13.0 [6.0, 20.0], *n* = 153	10.0 [5.0, 18.0], *n* = 99	12.0 [8.0, 19.0], *n* = 438
Pre-tracheostomy intubation, days	6.0 [4.0, 8.0], *n* = 835	5.0 [3.0, 8.0], *n* = 142	5.0 [4.0, 8.0], *n* = 153	8.0 [5.0, 8.0], *n* = 103	7.0 [4.0, 8.0], *n* = 437
Mechanical ventilation at rehabilitation admission, *n*/*N* (%)	184/862 (21.3)	10/148 (6.8)	36/152 (23.7)	18/107 (16.8)	120/455 (26.4)
Missing, *n* (%)	22 (2.5)	2 (1.3)	8 (5.0)	3 (2.7)	9 (1.9)
Early tracheostomy, *n*/*N* (%)	265/832 (31.9)	25/142 (17.6)	52/150 (34.7)	20/109 (18.3)	168/431 (39.0)
Missing, *n* (%)	52 (5.9)	8 (5.3)	10 (6.2)	1 (0.9)	33 (7.1)
Brainstem/posterior fossa involvement, *n*/*N* (%)	285/869 (32.8)	45/148 (30.4)	52/158 (32.9)	39/107 (36.4)	149/456 (32.7)
Missing, *n* (%)	15 (1.7)	2 (1.3)	2 (1.2)	3 (2.7)	8 (1.7)
Swallowing and airway-related characteristics					
Swallowing reflex present, *n*/*N* (%)	445/859 (51.8)	108/144 (75.0)	82/156 (52.6)	84/108 (77.8)	171/451 (37.9)
Missing, *n* (%)	25 (2.8)	6 (4.0)	4 (2.5)	2 (1.8)	13 (2.8)
Gag reflex present, *n*/*N* (%)	694/855 (81.2)	134/142 (94.4)	128/155 (82.6)	97/104 (93.3)	335/454 (73.8)
Missing, *n* (%)	29 (3.3)	8 (5.3)	5 (3.1)	6 (5.5)	10 (2.2)
Cough ability present, *n*/*N* (%)	438/858 (51.0)	96/144 (66.7)	76/156 (48.7)	59/110 (53.6)	207/448 (46.2)
Missing, *n* (%)	26 (2.9)	6 (4.0)	4 (2.5)	0 (0.0)	16 (3.4)
Suction frequency >2 times/8 h, *n*/*N* (%)	374/852 (43.9)	25/146 (17.1)	71/157 (45.2)	34/106 (32.1)	244/443 (55.1)
Missing, *n* (%)	32 (3.6)	4 (2.7)	3 (1.9)	4 (3.6)	21 (4.5)
Use of a privately hired caregiver, *n*/*N* (%)	793/875 (90.6)	143/149 (96.0)	148/157 (94.3)	106/109 (97.2)	396/460 (86.1)
Missing, *n* (%)	9 (1.0)	1 (0.7)	3 (1.9)	1 (0.9)	4 (0.9)

ICU, intensive care unit. Continuous variables are summarized using available observations. For categorical variables with missing data, percentages for observed categories use the non-missing denominator. Pre-tracheostomy intubation duration was coded as 0 days when no preceding endotracheal-intubation episode was documented. The median interval from neurological onset to rehabilitation admission was 27 [24, 30] days, and the median interval from tracheostomy to rehabilitation admission was 19 [17, 22] days. Among 865 patients with a preceding endotracheal-intubation episode, the median interval from neurological onset to intubation was 0 [0, 3] days; 72.3% were intubated within 0–1 day and only 7.3% at day 5 or later. Nineteen patients had no preceding endotracheal-intubation episode and were coded as 0 days for pre-tracheostomy intubation duration. Among patients with both variables observed, no patient classified as having early tracheostomy had more than 7 days of preceding endotracheal intubation. Detailed timing summaries and the early-tracheostomy cross-tabulation are provided in Supplementary material S4.

Within 30 days after rehabilitation admission, 574 patients (64.9%) underwent successful tracheostomy decannulation. The corresponding numbers were 143 of 150 (95.3%) with neither condition, 128 of 160 (80.0%) with pulmonary infection alone, 92 of 110 (83.6%) with nutritional risk alone, and 211 of 464 (45.5%) with both conditions. Ninety-eight patients were right-censored before day 30 without decannulation: 42 after acute-care or ICU transfer for clinical deterioration, 38 after transfer to another rehabilitation or long-term-care facility, and 18 after planned discharge with the tracheostomy tube still in place. No deaths occurred during observed rehabilitation follow-up. The Kaplan–Meier estimated 30-day probabilities of successful decannulation were 95.4%, 82.2%, 85.6%, and 49.5%, respectively. Median time to successful decannulation was 18.9 days (95% CI, 17.2–20.6) overall, 7.2 days (95% CI, 5.5–8.9) with neither condition, 11.0 days (95% CI, 9.1–15.1) with pulmonary infection alone, and 12.6 days (95% CI, 8.3–15.7) with nutritional risk alone; the median was not reached within 30 days in the dual-positive group. The cumulative curves and numbers at risk are shown in Figure 1 and Supplementary material S5.

**Figure 1:**
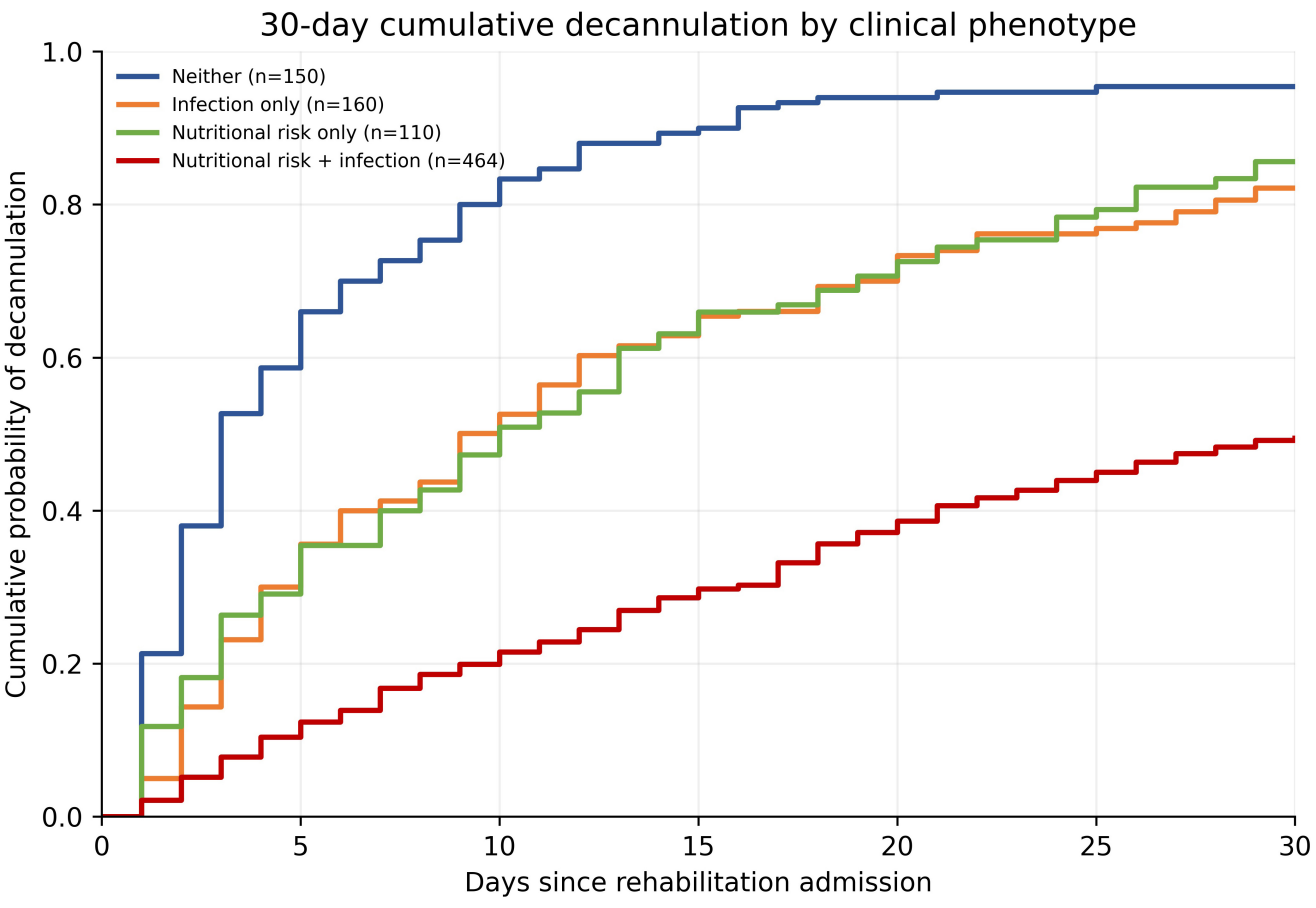
Cumulative probability of successful tracheostomy decannulation. Cumulative probability of successful tracheostomy decannulation according to nutritional risk and pulmonary infection phenotype. Time zero was admission to the rehabilitation department. Curves represent one minus the Kaplan–Meier survival estimate. Numbers at risk at days 0, 5, 10, 15, 20, 25, and 30 are reported in Supplementary material S5.

### 3.2 Primary Cox and exploratory analyses

Model 1 showed lower decannulation rates for pulmonary infection alone, nutritional risk alone, and both conditions. In the primary Model 2, pulmonary infection alone was associated with an HR of 0.619 (95% CI, 0.475–0.806; *P* < 0.001), nutritional risk alone with an HR of 0.642 (95% CI, 0.483–0.853; *P* = 0.002), and both conditions with an HR of 0.203 (95% CI, 0.157–0.262; *P* < 0.001). The multiplicative interaction HR was 0.510 (95% CI, 0.356–0.731; *P* < 0.001), and the likelihood-ratio test supported inclusion of the interaction term (χ^2^=13.17; *P* < 0.001). In the extended Model 3, estimates were similar: HRs were 0.642 (95% CI, 0.490–0.842; *P* = 0.001) for pulmonary infection alone, 0.665 (95% CI, 0.498–0.888; *P* = 0.006) for nutritional risk alone, 0.215 (95% CI, 0.164–0.283; *P* < 0.001) for both conditions, and 0.504 (95% CI, 0.350–0.727; *P* < 0.001) for the multiplicative interaction. Thus, the joint association was stronger than expected from the estimated individual associations on the multiplicative hazard scale. Exposure estimates are summarized in Table 2, with complete Model 2 and Model 3 coefficients in Supplementary materials S6, S7.

**Table 2:** Cox analysis of nutritional risk and pulmonary infection with tracheostomy decannulation outcomes.

Item	HR	95% CI	*P*
Model 1			
Infection only	0.421	0.330–0.535	< 0.001^***^
Nutritional risk only	0.441	0.338–0.574	< 0.001^***^
Both conditions	0.159	0.128–0.199	< 0.001^***^
Multiplicative interaction	0.859	0.777–0.950	0.003^**^
Model 2 (core–adjusted)			
Infection only	0.619	0.475–0.806	< 0.001^***^
Nutritional risk only	0.642	0.483–0.853	0.002^**^
Both conditions	0.203	0.157–0.262	< 0.001^***^
Multiplicative interaction	0.510	0.356–0.731	< 0.001^***^
Model 3 (extended)			
Infection only	0.642	0.490–0.842	0.001^**^
Nutritional risk only	0.665	0.498–0.888	0.006^**^
Both conditions	0.215	0.164–0.283	< 0.001^***^
Multiplicative interaction	0.504	0.350–0.727	< 0.001^***^

Model 2 is the primary core-adjusted model. Model 3 additionally includes concurrent respiratory-support, airway-protection, secretion-management, and care-related characteristics.

^**^P < 0.01, ^***^P < 0.001.

The magnitude of the multiplicative interaction varied across adjustment sets. In the crude model the interaction HR was 0.859, whereas a background-only model yielded an interaction HR of 1.199 and was not statistically significant; the interaction became more pronounced after adjustment for neurological severity and acute-care trajectory and remained similar after extended clinical adjustment (Supplementary material S8). This pattern indicates sensitivity of the interaction estimate to the adjustment set and argues against interpreting it as a model-invariant biological synergy.

### 3.3 Sensitivity and absolute-scale analyses

Standardized Model 2 estimates showed adjusted 30-day decannulation probabilities of 94.9% (95% CI, 82.2%−99.2%) with neither condition, 85.0% (64.5%−96.4%) with pulmonary infection alone, 86.0% (65.2%−96.7%) with nutritional risk alone, and 47.6% (30.8%−69.4%) with both conditions. The corresponding 30-day restricted mean times remaining tracheostomized were 10.5, 14.5, 14.2, and 22.8 days. The adjusted 30-day additive interaction contrast was −28.5 percentage points (95% CI, −35.2 to −15.8), indicating an adverse departure from additivity on the absolute probability scale. At 15 days, the corresponding interaction contrast was −16.4 percentage points (95% CI, −31.3 to −1.9). These absolute-scale results are shown in Table 3.

**Table 3:** Adjusted absolute decannulation outcomes by nutritional-risk–pulmonary-infection phenotype.

Phenotype	Adjusted 15–day decannulation probability	Adjusted 30–day decannulation probability	Restricted mean days remaining tracheostomized, 0–30 days
Neither	72.9% (53.3%−89.3%)	94.9% (82.2%−99.2%)	10.5
Pulmonary infection only	56.0% (36.1%−77.3%)	85.0% (64.5%−96.4%)	14.5
Nutritional risk only	57.3% (36.5%−78.3%)	86.0% (65.2%−96.7%)	14.2
Both conditions	24.0% (14.4%−40.1%)	47.6% (30.8%−69.4%)	22.8

Adjusted probabilities and restricted mean time remaining tracheostomized were standardized from primary Model 2. The 15-day additive interaction contrast was −16.4 percentage points (95% CI, −31.3 to −1.9), and the 30-day contrast was −28.5 percentage points (95% CI, −35.2 to −15.8). Negative values indicate an adverse departure from additivity in decannulation probability.

The 15-day Model 2 analysis was consistent with the primary analysis: HRs were 0.639 (95% CI, 0.473–0.864; *P* = 0.004) for pulmonary infection alone, 0.594 (95% CI, 0.425–0.828; *P* = 0.002) for nutritional risk alone, 0.173 (95% CI, 0.127–0.237; *P* < 0.001) for both conditions, and 0.457 (95% CI, 0.295–0.707; *P* < 0.001) for the interaction (Supplementary material S9). Adding neurological-onset-to-rehabilitation and tracheostomy-to-rehabilitation intervals to Model 2 produced similar estimates, including an interaction HR of 0.496 (95% CI, 0.345–0.713; *P* < 0.001; Supplementary material S4). Under the sensitivity assumption that every patient censored before day 30 remained non-decannulated through day 30, the interaction HR was 0.499 (95% CI, 0.348–0.715; *P* < 0.001) and the derived HR for both conditions was 0.199 (95% CI, 0.153–0.258; *P* < 0.001; Supplementary material S10). As censoring was disproportionately concentrated in the dual-positive phenotype, this assumption was not interpreted as conservative for the between-phenotype association. Excluding the 19 patients without a preceding endotracheal-intubation episode yielded nearly identical estimates: HR 0.614 (95% CI, 0.470–0.802; *P* < 0.001) for pulmonary infection alone, 0.640 (95% CI, 0.481–0.851; *P* = 0.002) for nutritional risk alone, 0.203 (95% CI, 0.157–0.263; *P* < 0.001) for both conditions, and 0.517 (95% CI, 0.360–0.742; *P* < 0.001) for the multiplicative interaction.

Scaled Schoenfeld residual tests showed no evidence of non-proportionality for nutritional risk, pulmonary infection, or their interaction. Time dependence was detected for ICU length of stay and Barthel Index; however, a time-split sensitivity analysis allowing these two coefficients to differ after day 15 yielded materially unchanged exposure estimates (nutritional risk HR, 0.627; pulmonary infection HR, 0.603; interaction HR, 0.518; derived both-conditions HR, 0.196; Supplementary material S11). The source review showed that pulmonary infection resulted in a higher applicable NRS-2002 disease-severity category in 24 of 624 patients with pulmonary infection (3.8%). In 10 patients, this difference was sufficient to change the dichotomized NRS-2002 classification at the ≥3 threshold. When the nutritional exposure was instead defined solely by an impaired-nutritional-status component score ≥1, the adjusted HRs were 0.628 (95% CI, 0.482–0.819; *P* = 0.001) for pulmonary infection alone, 0.671 (95% CI, 0.506–0.889; *P* = 0.006) for nutritional-status impairment alone, and 0.221 (95% CI, 0.170–0.287; *P* < 0.001) for both conditions. The multiplicative interaction remained present (HR, 0.525; 95% CI, 0.367–0.751; *P* < 0.001; Supplementary material S12).

### 3.4 Subgroup analyses

Exploratory subgroup contrasts showed the dual-positive phenotype was associated with a lower decannulation rate in each disease category: hemorrhagic stroke, HR 0.329 (95% CI, 0.254–0.427); ischemic stroke, 0.223 (0.130–0.383); traumatic brain injury, 0.110 (0.056–0.217); and other neurological conditions, 0.236 (0.154–0.363), with no clear evidence of heterogeneity (*P* = 0.513; Supplementary material S13). The corresponding HRs across admission GCS strata were 0.323 (0.251–0.416) for GCS 3–8, 0.311 (0.207–0.466) for GCS 9–12, and 0.188 (0.111–0.319) for GCS 13–15, with no evidence of heterogeneity (*P* = 0.380; Supplementary material S14). These exploratory contrasts describe the consistency of the dual-positive phenotype and are not subgroup-specific tests of the formal nutritional-risk-by-infection interaction.

## 4 Discussion

This study showed that nutritional risk and active pulmonary infection at rehabilitation admission were each associated with a lower rate of successful decannulation, and the lowest 30-day probability was observed when both conditions were present. The primary core-adjusted model and the extended clinical model yielded similar exposure estimates. The multiplicative interaction was statistically significant in both models, although its magnitude varied across adjustment sets and was not significant in the background-only model. Therefore, the interaction should be interpreted as an adjusted, model-dependent association rather than as evidence of biological synergy. The absolute-scale analysis pointed in the same clinical direction: the dual-positive phenotype had the longest restricted mean time remaining tracheostomized and an adverse departure from additivity in 30-day decannulation probability.

Previous studies have mainly examined neurological and respiratory readiness for decannulation. Pulmonary infection has repeatedly been identified as an unfavorable clinical condition, and evidence from neurological early rehabilitation and patients with disorders of consciousness supports its relevance to decannulation outcomes (19, 21). A recent systematic review reached a similar conclusion (10). Nutritional risk is also common during subacute rehabilitation after acquired brain injury and stroke (6, 34). Our findings extend this evidence by showing that a standardized nutritional-risk screening phenotype was associated with time to successful decannulation both alone and jointly with pulmonary infection.

Several mechanisms may plausibly link nutritional risk with delayed decannulation. NRS-2002 incorporates impaired nutritional status, disease severity, and age rather than measuring body weight alone (8). Patients entering neurological rehabilitation may have reduced intake because of dysphagia, impaired consciousness, or feeding dependence, while trauma, inflammation, and metabolic stress may increase nutritional requirements (6, 35–37). Nutritional risk may therefore coexist with reduced functional and muscle reserve. Previous studies have linked poor nutritional status and sarcopenia-related states with reduced expiratory muscle performance and impaired cough effectiveness, both of which are relevant to secretion clearance (9, 38, 39). However, muscle mass, respiratory muscle strength, and respiratory sarcopenia were not directly measured in this study; these pathways should therefore be regarded as possible explanations rather than observed mediators.

Pulmonary infection may have a more immediate relationship with decannulation readiness. Pneumonia has been associated with longer time to decannulation after severe traumatic brain injury (14) and recurrent pulmonary infection and secretion burden have been reported as barriers to tracheostomy removal (40). Infection may increase secretions, oxygen requirements, and the risk of respiratory deterioration, thereby reducing tolerance of capping and delaying progression through decannulation readiness assessment (1). Importantly, active infection was not an automatic permanent contraindication in our center. Adequate infection control and respiratory stability were considered together with airway patency, cough effectiveness, secretion management, oxygenation, and capping tolerance. Consequently, the present HRs should be interpreted as associations of infection status at rehabilitation admission with the rate of decannulation under routine clinical care, not as a purely biological causal effect of infection on eventual decannulation failure.

The joint association was the central finding. In the primary adjusted model, the dual-positive phenotype had a substantially lower decannulation rate than either condition alone, and the interaction indicated a joint association stronger than expected from the estimated individual associations on the multiplicative hazard scale. Standardized absolute outcomes supported the same clinical pattern: the dual-positive group had an adjusted 30-day decannulation probability of 47.6%, a restricted mean 22.8 days remaining tracheostomized over the 30-day window, and an additive interaction contrast of −28.5 percentage points. One possible explanation is that infection-related inflammation and reduced intake may worsen nutritional vulnerability, while limited nutritional and muscle reserve may impede cough and secretion clearance (15, 16). These mechanisms were not directly measured, and the interaction estimate changed across adjustment sets. As pulmonary infection contributed to the NRS-2002 disease-severity category in a small subset of patients, the primary exposures were not completely independent in their construction. However, a sensitivity analysis based solely on the impaired-nutritional-status component produced similar estimates, reducing concern that the observed joint association and interaction were explained solely by definitional overlap. The findings therefore support considering nutritional and respiratory burden together but do not establish a self-reinforcing biological mechanism or causality.

A strength of this study was its time-to-event design, direct analysis of the joint exposure, and complementary reporting on both multiplicative and absolute probability scales. The timing-adjusted, 15-day, censoring, and time-varying-coefficient sensitivity analyses produced similar exposure estimates. Exploratory disease and consciousness subgroup contrasts also showed a consistent direction. Nutritional risk and active pulmonary infection can both be identified at rehabilitation admission, and their coexistence may identify patients who warrant closer nutritional, respiratory, secretion-management, and airway-readiness assessment. Still, this study evaluated prognosis rather than treatment; the results do not show that nutritional intervention or infection treatment will shorten time to decannulation.

Several limitations should be acknowledged. First, this was a retrospective study from a single rehabilitation center, and residual confounding and limited generalizability remain possible. Although pre-rehabilitation timing was addressed in sensitivity analysis, detailed acute-care trajectories could not be fully reconstructed. Second, NRS-2002 is a screening instrument for nutritional risk rather than a diagnostic measure of malnutrition. Detailed nutrient intake, muscle mass, respiratory muscle strength, and formal sarcopenia measures were unavailable, and the binary exposure did not capture the type or severity of nutritional risk. Third, pulmonary infection was analyzed as a fixed baseline exposure. Detailed longitudinal information on infection severity, antimicrobial treatment, exact resolution time, persistence, and recurrence was not consistently available, particularly in earlier records. Because infection control formed one component of decannulation readiness, the infection estimate may reflect both baseline respiratory burden and subsequent clinical management. Fourth, discharge or transfer before decannulation could produce informative censoring. Estimates were stable under a sensitivity assumption that censored patients remained non-decannulated through day 30. However, censoring was disproportionately concentrated in the dual-positive phenotype, so this assumption could strengthen rather than attenuate between-phenotype contrasts. This analysis does not eliminate the possibility of differential informative censoring. Fifth, the multiplicative interaction was sensitive to the adjustment set; although the primary, extended, and absolute-scale analyses were concordant, the interaction should not be interpreted as a model-invariant biological synergy. Finally, subgroup contrasts were exploratory and less precise than the primary analysis. The “other neurological conditions” category also comprised several clinically distinct disorders with potentially different decannulation trajectories, which limits the interpretation and generalizability of estimates for this subgroup.

## Data Availability

The datasets presented in this article are not readily available because they contain clinical information subject to institutional ethics and privacy restrictions, but de-identified data may be obtained from the corresponding author upon reasonable request. Requests to access the datasets should be directed to Jing Wang, wangjiangjs@163.com.
