# Supplementary material for: Joint and interactive associations of nutritional risk and pulmonary infection with tracheostomy decannulation in neurorehabilitation inpatients: a retrospective cohort study
Source: Front Nutr. 2026 Oct 7;13:1944451. doi: 10.3389/fnut.2026.1944451 (PMC13683070; doi:10.3389/fnut.2026.1944451)
Supplement: Supplementary file 1 [file Supplementary_file_1.docx]

**Joint and Interactive Associations of Nutritional Risk and Pulmonary Infection With Tracheostomy Decannulation in Neurorehabilitation Inpatients: A Retrospective Cohort Study**

Supplementary Materials

Supplementary Material S1. Sample size estimation

Supplementary Material S2. Assessment, definitions, and rationale for covariate selection

Supplementary Material S3. Pre-imputation missingness by exposure phenotype

Supplementary Material S4. Pre-rehabilitation timing and early-tracheostomy consistency analyses

Supplementary Material S5. Numbers at risk for Figure 1

Supplementary Material S6. Complete coefficients for primary Model 2

Supplementary Material S7. Complete coefficients for extended Model 3

Supplementary Material S8. Sequential adjustment of exposure and interaction estimates

Supplementary Material S9. Fifteen-day core-adjusted sensitivity model

Supplementary Material S10. Censoring sensitivity analysis under an unfavorable-outcome assumption

Supplementary Material S11. Proportional-hazards diagnostics and time-varying-coefficient sensitivity

Supplementary Material S12. NRS-2002 component distributions and component-based sensitivity analysis

Supplementary Material S13. Disease-specific dual-positive phenotype contrasts

Supplementary Material S14. Glasgow Coma Scale (GCS)-stratified dual-positive phenotype contrasts

Abbreviations: CI, confidence interval; ETI, endotracheal intubation; GCS, Glasgow Coma Scale; HR, hazard ratio; ICU, intensive care unit; SE, standard error.

Supplementary Material S1. Sample size estimation

The sample size was estimated for the primary multiplicative interaction between nutritional risk and pulmonary infection in a Cox proportional hazards model. The calculation followed the method of Schmoor et al. for interaction effects between two binary covariates and was implemented using the ssizeEpiInt.default1 function in the R package powerSurvEpi. A two-sided α of 0.05 and a statistical power of 80% were specified. A prospective multicenter study of 831 tracheostomized patients undergoing neurological early rehabilitation reported a decannulation rate of approximately 57%. Therefore, the anticipated event proportion was set at 0.57. The prevalence of nutritional risk was set at 0.42 based on a study of patients with moderate-to-severe acquired brain injury admitted for subacute neurorehabilitation. The prevalence of pulmonary infection was set at 0.2685, and the association between hypoproteinemia and pulmonary infection was represented by an odds ratio of 1.922, based on a cohort of tracheostomized patients with severe traumatic brain injury. These estimates yielded expected joint exposure proportions of 0.4558, 0.1242, 0.2757, and 0.1443 for neither condition, pulmonary infection only, nutritional risk only, and both conditions, respectively. A minimum interaction hazard ratio of 0.55 was prespecified as a clinically relevant interaction effect. Under these assumptions, at least 802 evaluable patients were required. Allowing for approximately 5% of records with unavailable or irreconcilable key information increased the target number of screened records to 845. The final cohort of 884 patients therefore exceeded the estimated minimum sample-size requirement.

Supplementary Material S2. Assessment, definitions, and rationale for covariate selection

Adjustment variables were organized according to temporal and clinical role. The primary core adjustment set was intended to account for demographic characteristics, comorbidity, neurological severity, anatomical involvement, and acute-care trajectory. Concurrent respiratory-support, airway-protection, secretion-management, and care-related variables were reserved for the extended clinical model because they could reflect ongoing decannulation readiness rather than acting solely as baseline confounders. Unless otherwise specified, variables were obtained from medical records or standardized assessments completed at or before rehabilitation admission.

Age. Age was calculated in completed years at rehabilitation admission and was analyzed as a continuous variable. Age was included because older patients may have reduced respiratory reserve, cough strength, immune function, and rehabilitation potential, all of which may influence both pulmonary infection and the probability of successful decannulation.

Sex. Sex was obtained from the medical record and categorized as male or female. Sex was included because differences in body composition, respiratory muscle strength, underlying disease patterns, and smoking exposure may be associated with nutritional status, pulmonary complications, and decannulation outcomes.

Smoking history. Smoking history was determined from the admission medical record and categorized as present or absent. A positive smoking history included current or previous regular smoking over 6 months. Smoking was considered because it may impair pulmonary function, mucociliary clearance, and respiratory reserve and may therefore increase secretion burden and reduce the likelihood of successful decannulation.

Disease category. The primary diagnosis was categorized as hemorrhagic stroke, ischemic stroke, traumatic brain injury, or other neurological conditions. Disease category was used as a stratification factor to allow the baseline hazard to differ across underlying neurological conditions. The composition of the other neurological conditions group is shown below.

| **Clinical subgroup** | **n** | **%** | **Representative diagnoses** |
| --- | --- | --- | --- |
| Hypoxic–ischemic or post-resuscitation brain injury | 41 | 23.6 | Hypoxic–ischemic encephalopathy; post-cardiac-arrest brain injury |
| Spinal cord disorders and injuries | 53 | 30.5 | Cervical or thoracic spinal cord injury; traumatic spinal cord injury; spinal cord infarction or other vascular myelopathy; other spinal cord disorders |
| Central nervous system infectious, inflammatory, or demyelinating disorders | 18 | 10.3 | Viral encephalitis; autoimmune encephalitis; encephalomyelitis; acute disseminated encephalomyelitis; other inflammatory demyelinating disorders |
| Peripheral nerve or neuromuscular disorders | 6 | 3.4 | Guillain–Barré syndrome; other severe peripheral nerve or neuromuscular disorders |
| Other intracranial structural or neoplastic conditions | 19 | 10.9 | Hydrocephalus; intracranial tumors; postoperative intracranial structural lesions |
| Mixed or other cerebrovascular neurological conditions | 9 | 5.2 | Mixed ischemic and hemorrhagic cerebrovascular lesions; other cerebrovascular conditions not classifiable as isolated ischemic or hemorrhagic stroke |
| Other neurological disorders | 28 | 16.1 | Epilepsy-related neurological disorders; movement disorders; neurodegenerative disorders; other acquired neurological conditions |
| **Total** | **174** | **100.0** | — |

Charlson Comorbidity Index. The Charlson Comorbidity Index was calculated from comorbid conditions documented in the admission medical record using the conventional weighted scoring system and was analyzed as a continuous variable. It was included as an indicator of chronic disease burden because multimorbidity may affect nutritional status, susceptibility to infection, physiological reserve, and the capacity to tolerate decannulation.

Admission Glasgow Coma Scale. The Glasgow Coma Scale score recorded within 24 h of rehabilitation admission was used and ranged from 3 to 15, with higher scores indicating a higher level of consciousness. The score was included because impaired consciousness may reduce spontaneous airway protection, the ability to follow commands, cough effectiveness, and the management of oral and respiratory secretions.

Barthel Index. Activities of daily living were assessed using the Barthel Index within 24 h of admission. The total score ranged from 0 to 100, with higher scores indicating greater functional independence. The Barthel Index was included as a measure of global functional impairment and rehabilitation potential, which may reflect mobility, respiratory muscle activity, self-care ability, and the capacity to participate in airway-clearance training.

Intensive care unit (ICU) length of stay. ICU length of stay was defined as the cumulative number of days spent in an intensive care unit during the index acute-care episode before rehabilitation admission and was analyzed continuously per 7-day increase. A prolonged ICU stay may reflect greater acute illness burden, immobilization, infection exposure, sedation, and respiratory muscle deconditioning. During the reviewer-requested data-quality audit, source-specific ICU coding was harmonized to this single definition before the revised analyses were performed. Source verification resulted in modification of ICU length-of-stay values in 296 of 884 patients (33.5%). The predominant issue was incomplete aggregation of ICU duration across units or episodes. A smaller number of records contained overlapping or duplicated episode-level durations. Reconstructed values were verified against ICU admission/discharge and transfer records, and all descriptive and model-based analyses involving ICU length of stay were rerun using the reconstructed variable.

Pre-tracheostomy intubation duration. This variable was defined as the number of days between the endotracheal-intubation episode immediately preceding tracheostomy and the tracheostomy procedure. Patients without a preceding endotracheal-intubation episode were coded as 0 days. The variable was analyzed continuously per 7-day increase and was not truncated or categorized in the primary Cox models.

Mechanical ventilation. Mechanical ventilation was categorized as present or absent according to whether invasive ventilatory support remained necessary at rehabilitation admission. Because this measure reflects concurrent respiratory-support needs and is closely related to decannulation readiness, it was included in the extended Model 3.

Early tracheostomy. Early tracheostomy was defined as tracheostomy performed within 7 days after the onset of the neurological lesion or traumatic brain injury. Tracheostomy performed more than 7 days after onset was classified as non-early tracheostomy. Timing of tracheostomy was included because it may reflect acute-care management, duration of endotracheal intubation, underlying disease severity, and the subsequent course of airway rehabilitation.

Pre-rehabilitation timing variables. Neurological-onset-to-rehabilitation was calculated as the interval from onset of the current neurological condition to rehabilitation admission. Tracheostomy-to-rehabilitation was calculated from tracheostomy to rehabilitation admission. These intervals were not included in the primary model but were added together in a timing sensitivity analysis to address differences in pre-rehabilitation disease and airway trajectory.

Brainstem or posterior fossa involvement. Brainstem or posterior fossa involvement was determined from the primary diagnosis and available neuroimaging reports and was categorized as present or absent. This variable was included because lesions in these regions may directly affect respiratory control, cranial nerve function, swallowing, cough generation, and airway-protective reflexes.

Swallowing reflex. Swallowing reflex was assessed according to the patient’s level of consciousness. Patients able to cooperate were asked to swallow saliva or a small amount of water, while hyoid and laryngeal elevation was observed or palpated. In patients with impaired consciousness, spontaneous saliva swallowing was observed for 3–5 min; if absent, approximately 0.4 mL of sterile water was instilled into the pharynx. The reflex was considered present when a definite swallowing movement occurred spontaneously or within 3 s of stimulation. It was included because impaired initiation of pharyngeal swallowing may reduce secretion clearance and affect pulmonary infection and decannulation.

Gag reflex. The gag reflex was assessed by gently stimulating the soft palate or posterior pharyngeal wall with a cotton swab. The reflex was considered present when stimulation elicited soft-palate elevation, pharyngeal-wall contraction, or an observable gag response; otherwise, it was considered absent. The gag reflex was included as a clinical indicator of the integrity of the pharyngeal sensory–motor reflex arc, which may be related to airway protection and secretion management.

Cough ability. Cough ability was assessed using the white card test. A white card or piece of gauze was placed immediately in front of the tracheostomy tube opening. Patients who were able to follow commands were instructed to cough, whereas spontaneous or reflex coughing was observed in those unable to cooperate. Cough ability was considered present when airway secretions were expelled onto the card or gauze and absent when the patient was unable to expel secretions to the tracheostomy tube opening. Cough ability was included because it provides a simple bedside assessment of the ability to generate an effective cough and clear airway secretions, which may influence pulmonary infection, tolerance of tracheostomy tube capping, and successful decannulation.

Suctioning frequency. Suctioning frequency was obtained from nursing and airway-management records during the initial assessment period. Patients were categorized according to whether tracheal suctioning was required more than twice within an 8-h period. A frequency of more than two suctioning episodes per 8 h indicated a high secretion burden or inadequate spontaneous clearance. This variable was included because dependence on frequent suctioning may increase the risk of airway obstruction and respiratory deterioration after decannulation.

Dedicated caregiver. Dedicated caregiver use was categorized as present or absent according to whether a privately hired caregiver was assigned at rehabilitation admission to provide regular bedside assistance in addition to routine clinical care. Such assistance commonly included repositioning, back percussion, oral care, observation of secretions, and support with feeding and daily activities. Dedicated caregiver use was included because continuity and intensity of bedside care may influence nutritional delivery, secretion clearance, pulmonary infection control, and participation in rehabilitation.

Supplementary Material S3. Pre-imputation missingness by exposure phenotype

Key exposures (nutritional risk and pulmonary infection) and the decannulation outcome were complete in the final cohort. Remaining covariate missingness before multiple imputation is summarized below. No individual covariate exceeded 7% missingness overall.

| **Variable** | **Overall n (%)** | **Neither n (%)** | **Infection only n (%)** | **Nutritional risk only n (%)** | **Both n (%)** |
| --- | --- | --- | --- | --- | --- |
| Age | 0 (0.0) | 0 (0.0) | 0 (0.0) | 0 (0.0) | 0 (0.0) |
| Sex | 0 (0.0) | 0 (0.0) | 0 (0.0) | 0 (0.0) | 0 (0.0) |
| Smoking history | 11 (1.2) | 3 (2.0) | 1 (0.6) | 2 (1.8) | 5 (1.1) |
| Charlson Comorbidity Index | 13 (1.5) | 3 (2.0) | 1 (0.6) | 4 (3.6) | 5 (1.1) |
| Glasgow Coma Scale | 8 (0.9) | 0 (0.0) | 2 (1.2) | 2 (1.8) | 4 (0.9) |
| Barthel Index | 20 (2.3) | 4 (2.7) | 2 (1.2) | 2 (1.8) | 12 (2.6) |
| Brainstem/posterior fossa involvement | 15 (1.7) | 2 (1.3) | 2 (1.2) | 3 (2.7) | 8 (1.7) |
| Pre-tracheostomy intubation duration | 49 (5.5) | 8 (5.3) | 7 (4.4) | 7 (6.4) | 27 (5.8) |
| ICU length of stay | 58 (6.6) | 14 (9.3) | 7 (4.4) | 11 (10.0) | 26 (5.6) |
| Mechanical ventilation | 22 (2.5) | 2 (1.3) | 8 (5.0) | 3 (2.7) | 9 (1.9) |
| Early tracheostomy | 52 (5.9) | 8 (5.3) | 10 (6.2) | 1 (0.9) | 33 (7.1) |
| Swallowing reflex | 25 (2.8) | 6 (4.0) | 4 (2.5) | 2 (1.8) | 13 (2.8) |
| Gag reflex | 29 (3.3) | 8 (5.3) | 5 (3.1) | 6 (5.5) | 10 (2.2) |
| Cough ability | 26 (2.9) | 6 (4.0) | 4 (2.5) | 0 (0.0) | 16 (3.4) |
| Suction frequency | 32 (3.6) | 4 (2.7) | 3 (1.9) | 4 (3.6) | 21 (4.5) |
| Privately hired caregiver | 9 (1.0) | 1 (0.7) | 3 (1.9) | 1 (0.9) | 4 (0.9) |

Supplementary Material S4. Pre-rehabilitation timing and early-tracheostomy consistency analyses

| **Interval** | **Overall** | **Neither** | **Infection only** | **Nutritional risk only** | **Both** |
| --- | --- | --- | --- | --- | --- |
| Neurological onset → rehabilitation, days | 27 [24, 30] | 26 [24, 28] | 28 [25, 30] | 27 [24, 30] | 28 [25, 31] |
| Tracheostomy → rehabilitation, days | 19 [17, 22] | 18 [16, 20] | 20 [18, 22] | 19 [16, 21] | 20 [18, 22] |
| Neurological onset → tracheostomy, days | 8 [6, 9] | 8 [8, 9] | 8 [5, 9] | 8 [8, 9] | 8 [5, 9] |

Among 865 patients with a preceding endotracheal-intubation episode, the median neurological-onset-to-intubation interval was 0 [0, 3] days; 72.3% were intubated within 0–1 day and 7.3% at day 5 or later. Nineteen patients had no preceding endotracheal-intubation episode and were coded as 0 days for the pre-tracheostomy intubation variable. ETI denotes endotracheal intubation. The cross-tabulation was restricted to the 814 patients with both early-tracheostomy status and pre-tracheostomy intubation duration observed. Of the remaining 70 patients, 31 had both variables missing, 21 had missing early-tracheostomy status only, and 18 had missing pre-tracheostomy intubation duration only. Missing observations were not assigned to any cross-tabulation category. Of the 19 patients without a preceding ETI, 17 had observed early-tracheostomy status and are represented in this table.

| **Early-tracheostomy status** | **No preceding ETI (0 days)** | **ETI→tracheostomy 1–7 days** | **ETI→tracheostomy >7 days** |
| --- | --- | --- | --- |
| Early tracheostomy | 11 | 247 | 0 |
| Non-early tracheostomy | 6 | 224 | 326 |
| Total | 17 | 471 | 326 |
| **Association** | **Timing-adjusted HR** | **95% CI** | **P** |
| Nutritional risk only | 0.659 | 0.493–0.881 | 0.005 |
| Pulmonary infection only | 0.626 | 0.479–0.817 | <0.001 |
| Multiplicative interaction | 0.496 | 0.345–0.713 | <0.001 |
| Both conditions | 0.205 | 0.157–0.266 | <0.001 |

Timing-adjusted Model 2 additionally included neurological-onset-to-rehabilitation and tracheostomy-to-rehabilitation intervals, each per 7-day increase.

Supplementary Material S5. Numbers at risk for Figure 1

| **Day** | **Overall** | **Neither** | **Infection only** | **Nutritional risk only** | **Both** |
| --- | --- | --- | --- | --- | --- |
| 0 | 884 | 150 | 160 | 110 | 464 |
| 5 | 735 | 94 | 120 | 88 | 433 |
| 10 | 588 | 57 | 87 | 58 | 386 |
| 15 | 480 | 33 | 68 | 47 | 332 |
| 20 | 381 | 19 | 51 | 36 | 275 |
| 25 | 308 | 11 | 35 | 25 | 237 |
| 30 | 212 | 5 | 22 | 13 | 172 |

Supplementary Material S6. Complete coefficients for primary Model 2

| **Variable** | **β** | **SE** | **HR** | **95% CI** | **P** |
| --- | --- | --- | --- | --- | --- |
| Nutritional risk | -0.443 | 0.144 | 0.642 | 0.483–0.853 | 0.002 |
| Pulmonary infection | -0.480 | 0.134 | 0.619 | 0.475–0.806 | <0.001 |
| Nutritional risk × pulmonary infection | -0.672 | 0.183 | 0.510 | 0.356–0.731 | <0.001 |
| Both conditions (derived) | -1.596 | 0.131 | 0.203 | 0.157–0.262 | <0.001 |
| Age per 10 years | -0.006 | 0.031 | 0.994 | 0.934–1.057 | 0.845 |
| Male sex | -0.107 | 0.098 | 0.898 | 0.741–1.089 | 0.274 |
| Smoking history | -0.012 | 0.098 | 0.988 | 0.814–1.199 | 0.902 |
| Charlson Comorbidity Index | -0.054 | 0.046 | 0.947 | 0.865–1.037 | 0.242 |
| Glasgow Coma Scale | -0.011 | 0.016 | 0.989 | 0.958–1.021 | 0.486 |
| Barthel Index per 10 points | 0.130 | 0.027 | 1.140 | 1.079–1.204 | <0.001 |
| Brainstem/posterior fossa involvement | 0.053 | 0.089 | 1.055 | 0.886–1.256 | 0.551 |
| Pre-tracheostomy intubation duration per 7 days | 0.164 | 0.108 | 1.179 | 0.954–1.457 | 0.128 |
| ICU length of stay per 7 days | -0.093 | 0.030 | 0.911 | 0.858–0.967 | 0.002 |
| Early tracheostomy | 0.084 | 0.106 | 1.088 | 0.882–1.341 | 0.431 |

Model 2 was stratified by disease category. The both-conditions estimate was derived from the sum of the nutritional-risk, pulmonary-infection, and product-term coefficients.

Supplementary Material S7. Complete coefficients for extended Model 3

| **Variable** | **β** | **SE** | **HR** | **95% CI** | **P** |
| --- | --- | --- | --- | --- | --- |
| Nutritional risk | -0.408 | 0.147 | 0.665 | 0.498–0.888 | 0.006 |
| Pulmonary infection | -0.443 | 0.138 | 0.642 | 0.490–0.842 | 0.001 |
| Nutritional risk × pulmonary infection | -0.684 | 0.186 | 0.504 | 0.350–0.727 | <0.001 |
| Both conditions (derived) | -1.536 | 0.139 | 0.215 | 0.164–0.283 | <0.001 |
| Age per 10 years | -0.005 | 0.031 | 0.994 | 0.935–1.058 | 0.858 |
| Male sex | -0.102 | 0.099 | 0.903 | 0.743–1.096 | 0.301 |
| Smoking history | -0.025 | 0.099 | 0.974 | 0.802–1.183 | 0.793 |
| Charlson Comorbidity Index | -0.060 | 0.046 | 0.941 | 0.859–1.031 | 0.193 |
| Glasgow Coma Scale | -0.035 | 0.019 | 0.965 | 0.928–1.003 | 0.070 |
| Barthel Index per 10 points | 0.127 | 0.028 | 1.136 | 1.075–1.201 | <0.001 |
| Brainstem/posterior fossa involvement | 0.038 | 0.089 | 1.039 | 0.872–1.239 | 0.667 |
| Pre-tracheostomy intubation duration per 7 days | 0.150 | 0.108 | 1.162 | 0.940–1.437 | 0.164 |
| ICU length of stay per 7 days | -0.082 | 0.033 | 0.921 | 0.863–0.983 | 0.013 |
| Early tracheostomy | 0.067 | 0.107 | 1.070 | 0.867–1.321 | 0.530 |
| Mechanical ventilation at rehabilitation admission | -0.021 | 0.122 | 0.979 | 0.770–1.243 | 0.859 |
| Swallowing reflex present | 0.107 | 0.100 | 1.113 | 0.914–1.356 | 0.286 |
| Gag reflex present | -0.002 | 0.122 | 0.998 | 0.785–1.268 | 0.985 |
| Cough ability present | 0.218 | 0.094 | 1.244 | 1.034–1.498 | 0.021 |
| Suction frequency >2/8h | -0.062 | 0.094 | 0.939 | 0.780–1.131 | 0.507 |
| Privately hired caregiver | -0.020 | 0.159 | 0.980 | 0.717–1.338 | 0.898 |

Model 3 included all Model 2 variables plus mechanical ventilation at rehabilitation admission, swallowing reflex, gag reflex, cough ability, suction frequency, and privately hired caregiver.

Supplementary Material S8. Sequential adjustment of exposure and interaction estimates

| **Association** | **Model 1 crude** | **Background-only Model A** | **Model 2 core-adjusted** | **Model 3 extended** |
| --- | --- | --- | --- | --- |
| Pulmonary infection only | 0.421 (0.330–0.535) | 0.440 (0.344–0.563) | 0.619 (0.475–0.806) | 0.642 (0.490–0.842) |
| Nutritional risk only | 0.441 (0.338–0.574) | 0.385 (0.292–0.509) | 0.642 (0.483–0.853) | 0.665 (0.498–0.888) |
| Both conditions | 0.159 (0.128–0.199) | 0.203 (0.162–0.255) | 0.203 (0.157–0.262) | 0.215 (0.164–0.283) |
| Multiplicative interaction | 0.859 (0.777–0.950) | 1.199 (0.835–1.720) | 0.510 (0.356–0.731) | 0.504 (0.350–0.727) |
| Interaction P | 0.003 | 0.324 | <0.001 | <0.001 |

Background-only Model A adjusted for age, sex, smoking history, and Charlson Comorbidity Index and was stratified by disease category. The interaction estimate was sensitive to the adjustment set and became more pronounced after neurological and acute-care characteristics were accounted for.

Supplementary Material S9. Fifteen-day core-adjusted sensitivity model

| **Variable** | **β** | **SE** | **HR** | **95% CI** | **P** |
| --- | --- | --- | --- | --- | --- |
| Nutritional risk | -0.521 | 0.169 | 0.594 | 0.425–0.828 | 0.002 |
| Pulmonary infection | -0.447 | 0.153 | 0.639 | 0.473–0.864 | 0.004 |
| Nutritional risk × pulmonary infection | -0.783 | 0.222 | 0.457 | 0.295–0.707 | <0.001 |
| Both conditions (derived) | -1.752 | 0.159 | 0.173 | 0.127–0.237 | <0.001 |
| Age per 10 years | -0.000 | 0.038 | 0.999 | 0.927–1.078 | 0.986 |
| Male sex | -0.040 | 0.123 | 0.960 | 0.753–1.223 | 0.741 |
| Smoking history | -0.047 | 0.123 | 0.954 | 0.749–1.215 | 0.702 |
| Charlson Comorbidity Index | -0.035 | 0.056 | 0.966 | 0.865–1.078 | 0.533 |
| Glasgow Coma Scale | -0.007 | 0.020 | 0.992 | 0.954–1.032 | 0.699 |
| Barthel Index per 10 points | 0.083 | 0.034 | 1.087 | 1.016–1.162 | 0.016 |
| Brainstem/posterior fossa involvement | -0.066 | 0.113 | 0.936 | 0.750–1.169 | 0.559 |
| Pre-tracheostomy intubation duration per 7 days | 0.283 | 0.133 | 1.328 | 1.022–1.725 | 0.034 |
| ICU length of stay per 7 days | -0.155 | 0.040 | 0.856 | 0.791–0.927 | <0.001 |
| Early tracheostomy | 0.135 | 0.132 | 1.145 | 0.883–1.484 | 0.307 |

By day 15, 374 successful decannulation events had occurred: 117 in the neither group, 91 in the pulmonary-infection-only group, 62 in the nutritional-risk-only group, and 104 in the dual-positive group. Thirty patients were censored before day 15 (0, 1, 1, and 28 across the four groups, respectively), and the remaining 480 event-free patients were administratively censored at day 15.

Supplementary Material S10. Censoring sensitivity analysis under an unfavorable-outcome assumption

| **Phenotype** | Acute-care/ICU transfer | | Other rehabilitation/long-term-care transfer | | Planned discharge with tracheostomy | | Total pre-day-30 censors |
| --- | --- | --- | --- | --- | --- | --- | --- |
| Neither | 0 | | 1 | | 1 | | 2 |
| Infection only | 5 | | 3 | | 2 | | 10 |
| Nutritional risk only | 1 | | 2 | | 2 | | 5 |
| Both | 36 | | 32 | | 13 | | 81 |
| **Association** | | **Sensitivity-analysis HR** | | **95% CI** | | **P** | |
| Pulmonary infection only | | 0.617 | | 0.474–0.804 | | <0.001 | |
| Nutritional risk only | | 0.646 | | 0.486–0.858 | | 0.003 | |
| Both conditions | | 0.199 | | 0.153–0.258 | | <0.001 | |
| Multiplicative interaction | | 0.499 | | 0.348–0.715 | | <0.001 | |

In this sensitivity analysis, every patient censored before day 30 was retained as non-decannulated through day 30. This represents an unfavorable-outcome assumption for censored individuals but is not necessarily conservative for between-phenotype associations because 81 of the 98 pre-day-30 censors were in the dual-positive group. The slightly more extreme estimates therefore indicate stability under this specific assumption rather than attenuation toward the null. The analysis does not eliminate the possibility of differential informative censoring.

Supplementary Material S11. Proportional-hazards diagnostics and time-varying-coefficient sensitivity

| **Parameter** | **Scaled Schoenfeld test P** |
| --- | --- |
| Nutritional risk | 0.296 |
| Pulmonary infection | 0.451 |
| Nutritional risk × pulmonary infection | 0.401 |
| Barthel Index | 0.002 |
| ICU length of stay | <0.001 |

The primary exposure terms did not show evidence of non-proportional hazards. Time dependence was detected for Barthel Index and ICU length of stay. A time-split sensitivity model allowed the coefficients for these two adjustment variables to differ after day 15.

| **Association** | **HR** | **95% CI** | **P** |
| --- | --- | --- | --- |
| Nutritional risk only | 0.627 | 0.469–0.837 | 0.002 |
| Pulmonary infection only | 0.603 | 0.462–0.788 | <0.001 |
| Both conditions | 0.196 | 0.151–0.253 | <0.001 |
| Multiplicative interaction | 0.518 | 0.360–0.744 | <0.001 |

Supplementary Material S12. NRS-2002 component distributions and component-based sensitivity analysis

| NRS-2002 component | Neither, n=150 | Infection only, n=160 | Nutritional risk only, n=110 | Both, n=464 |
| --- | --- | --- | --- | --- |
| **Impaired nutritional status** | | | | |
| Score 0 | 145 (96.7%) | 153 (95.6%) | 4 (3.6%) | 15 (3.2%) |
| Score 1 | 5 (3.3%) | 7 (4.4%) | 48 (43.6%) | 169 (36.4%) |
| Score 2 | 0 | 0 | 45 (40.9%) | 196 (42.2%) |
| Score 3 | 0 | 0 | 13 (11.8%) | 84 (18.1%) |
| **Disease severity** | | | | |
| Score 0 | 4 (2.7%) | 2 (1.2%) | 0 | 0 |
| Score 1 | 35 (23.3%) | 30 (18.8%) | 15 (13.6%) | 49 (10.6%) |
| Score 2 | 111 (74.0%) | 128 (80.0%) | 85 (77.3%) | 360 (77.6%) |
| Score 3 | 0 | 0 | 10 (9.1%) | 55 (11.9%) |
| **Age point** | | | | |
| 0 | 145 (96.7%) | 154 (96.2%) | 91 (82.7%) | 364 (78.4%) |
| 1 | 5 (3.3%) | 6 (3.8%) | 19 (17.3%) | 100 (21.6%) |

| Association | Adjusted HR | 95% CI | P |
| --- | --- | --- | --- |
| Pulmonary infection only | 0.628 | 0.482–0.819 | 0.001 |
| Nutritional-status impairment only | 0.671 | 0.506–0.889 | 0.006 |
| Both | 0.221 | 0.170–0.287 | <0.001 |
| Multiplicative interaction | 0.525 | 0.367–0.751 | <0.001 |

Supplementary Material S13. Disease-specific dual-positive phenotype contrasts

| **Disease group** | **N** | **Adjusted HR** | **95% CI** | **P** |
| --- | --- | --- | --- | --- |
| Hemorrhagic stroke | 469 | 0.329 | 0.254–0.427 | <0.001 |
| Ischemic stroke | 126 | 0.223 | 0.130–0.383 | <0.001 |
| Traumatic brain injury | 115 | 0.110 | 0.056–0.217 | <0.001 |
| Other neurological conditions | 174 | 0.236 | 0.154–0.363 | <0.001 |

HRs compare the dual-positive phenotype with all other nutritional-risk–pulmonary-infection phenotypes. Global heterogeneity likelihood-ratio χ²(3)=2.30, P=0.513.

Supplementary Material S14. Glasgow Coma Scale (GCS)-stratified dual-positive phenotype contrasts

| **Admission GCS** | **N** | **Adjusted HR** | **95% CI** | **P** |
| --- | --- | --- | --- | --- |
| 3-8 | 546 | 0.323 | 0.251–0.416 | <0.001 |
| 9-12 | 220 | 0.311 | 0.207–0.466 | <0.001 |
| 13-15 | 118 | 0.188 | 0.111–0.319 | <0.001 |

HRs compare the dual-positive phenotype with all other phenotypes. Global heterogeneity likelihood-ratio χ²(2)=1.93, P=0.380.
